# Highly Efficient Genome Editing Using Geminivirus-Based CRISPR/Cas9 System in Cotton Plant

**DOI:** 10.3390/cells11182902

**Published:** 2022-09-16

**Authors:** Bo Li, Chunyang Fu, Jiawei Zhou, Fengjiao Hui, Qiongqiong Wang, Fuqiu Wang, Guanying Wang, Zhongping Xu, Lianlian Che, Daojun Yuan, Yanqin Wang, Xianlong Zhang, Shuangxia Jin

**Affiliations:** 1 Hubei Hongshan Laboratory, National Key Laboratory of Crop Genetic Improvement, Huazhong Agricultural University, Wuhan 430070, China; 2 Institute of Nuclear and Biological Technology, Xinjiang Academy of Agricultural Sciences/Xinjiang Key Laboratory of Crop Biotechnology, Urumqi 830091, China; 3 Xinjiang Production and Construction Corps Key Laboratory of Protection and Utilization of Biological Resources in Tarim Basin, Tarim University, Alaer 843300, China

**Keywords:** cotton plant, CRISPR/Cas9, gRNA, bean yellow dwarf virus (BeYDV) replicon, genome editing

## Abstract

Upland cotton (*Gossypium hirsutum*), an allotetraploid, contains At- and Dt- subgenome and most genes have multiple homologous copies, which pose a huge challenge to investigate genes’ function due to the functional redundancy. Therefore, it is of great significance to establish effective techniques for the functional genomics in cotton. In this study, we tested two novel genome editing vectors and compared them with the CRISPR/Cas9 system (pRGEB32-GhU6.7) developed in our laboratory previously. In the first new vector, the sgRNA transcription unite was constructed into the replicon (LIR-Donor-SIR-Rep-LIR) of the bean yellow dwarf virus (BeYDV) and named as pBeYDV-Cas9-KO and in the second vector, the ubiquitin promoter that drives Cas9 protein was replaced with a constitutive CaMV 35S promoter and defined as pRGEB32-35S. The results from transgenic cotton calli/plants revealed that pBeYDV-Cas9-KO vector showed the highest editing efficiency of *GhCLA1* in At and Dt subgenomes edited simultaneously up to 73.3% compared to the 44.6% of pRGEB32-GhU6.7 and 51.2% of pRGEB32-35S. The editing efficiency of *GhCLA1* in At and Dt subgenome by pBeYDV-Cas9-KO was 85.7% and 97.2%, respectively, whereas the efficiency by pRGEB32-GhU6.7 and pRGEB32-35S vectors was 67.7%, 86.5%, 84%, and 87.2%, respectively. The editing profile of pBeYDV-Cas9-KO was mainly composed of fragment deletion, accounting for 84.0% and ranging 1–10 bp in length. The main editing sites are located at positions 11–17 upstream of PAM site. The off-target effects were not detected in all potential off-target sites. Taken together, the pBeYDV-Cas9-KO system has high editing efficiency and specificity with wide editing range than the traditional CRISPR/Cas9 system, which provides a powerful tool for cotton functional genomics research and molecular breeding.

## 1. Introduction

CRISPR/Cas technology provides an effective tool for plant functional genomics and molecular breeding. At present, CRISPR/Cas gene editing technology has become a conventional molecular breeding method in major crop species such as rice, corn, wheat, soybean, oilseed rape, and other major plant species [1,2,3,4,5]. Polyploidy is a widespread phenomenon in angiosperms. Allopolyploid genomes are derived from combinations of genomes from different species [6]. More than 90% of fiber products come from allotetraploid cotton (*G. hirsutum* and *G. barbadense*), which originated from an allopolyplodization event approximately 1–2 million year ago, followed by millennia of asymmetric subgenome selection [7]. However, the editing efficiency of CRISPR/Cas9 in complex genomes (Ployploid) such as cotton is still low because multiple copies of homologous genes scattered in different homologous chromosomes, which resulted in higher ratio of heterozygous mutation when the CRISPR/Cas system was applied for genome editing [2,8,9,10,11,12]. Therefore, the development of efficient genome editing tools is still of great significance for functional genomics and molecular breeding in cotton.

CRISPR/Cas systems provide bacteria and archaea with adaptive immunity against viruses and plasmids. These defense systems rely on Cas proteins and gRNA to accurately detect and destroy foreign invading genetic materials [13,14,15]. CRISPR/Cas9 system belongs to Class II system and has been widely applied in eukaryotic cell genome editing [16,17], which is mainly composed of Cas9 nuclease protein and guide RNA (crRNA/tracRNA), and combines with the sequence of target sites to form a complex. Upon binding gRNA, the Cas9-gRNA complex identifies protospacer adjacent motif (PAM) sequences and induces DNA double-strand breaks (DSBs) at the targeted region complementary to crRNA. The resultant DSBs act as beacons for cellular repair machinery that introduces random insertions and/or deletions (indels) [16,18]. For the plant genome editing, CRISPR/Cas9 system is modified by means of plant specific promoter selection, codon optimization of Cas9 protein, and so on to specifically edit the genome sequences of plants. Then, CRISPR/Cas9 genome editing systems were successfully applied in Arabidopsis and rice first with high editing efficiency and specificity [19,20]. Since then, genome editing systems have been developed in many plant species. So far, the mutation induction efficiency of CRISPR/Cas9 system in rice can reach more than 80% [21,22,23,24]. The highest editing efficiency can reach 87% in maize [25]. In addition, CRISPR/Cas9 system has also been successfully applied in wheat [3,5,26,27,28], barley [29], cotton [2,8,10,30], tobacco [31,32], rape [33], tomato [34], rice [35], soybean [36,37,38], and other major crops with relatively lower editing efficiency due to the complex genomes of these species or/and low efficiency of genetic transformation system.

Providing sufficient gRNA and donor templates in the DNA double strands break (DSB) repairing can improve the efficiency of gene editing (knock in and knock out) [39,40]. Geminiviruses, characterized by rapid infection, replication, transcription, and expression without integration into plant genome [41], are suitable to be modified as vectors providing sufficient transcripts to improve gene editing efficiency in cells [4]. Geminiviruses constitute a large family of plant-infecting DNA viruses, which is mainly composed of one (monopartite) or two (bipartite) DNA components that encode 5–7 proteins involved in viral replication, movement, transmission, and pathogenesis [42]. Their genomes are composed of 2.5–3.0 kb single stranded circular DNA. We chose to use the mild strain of the bean yellow dwarf virus (BeYDV) because it was successfully used to express heterologous proteins in plant cells [43]. BeYDV replication requires three viral elements: the cis-acting long intergenic region (LIR), the short intergenic region (SIR), and the trans-acting replication initiation protein (Rep/RepA) [41,44]. By modifying the genome structure of geminivirus, the coding sequence of motion and coat protein were replaced by site-specific nuclease and gene targeted donor template, and a LIR was placed on each side of the virus genome to ensure release from T-DNA in plant cells. We introduced the replicon fragment of geminivirus BeYDV into the borders of a modified Ti plasmid vector of the Agrobacterium. Then we infected cotton by Agrobacterium mediated transformation with Ti plasmid containing the BeYDV elements which were inserted into the cotton genome by plant genetic transformation. During the Agrobacterium infection, linear T-DNA molecules are transported to the plant nucleus to produce geminivirus replicon in the cell [44,45,46]. Thus, a large number of gRNA and donor molecules can be produced in the cell by rolling-circle replication (replicon), and the number can be as high as hundreds to thousands of copies, so as to greatly improve the probability of gene editing. Co-delivery of pLSL T-DNA containing SSNs (sequence-specific nucleases) and repair templates with Rep/RepA can coordinate SSN expression, increase the copy number of repair templates in plant cell nucleus, and improve the gene targeting efficiency in tobacco [41]. However, this strategy has not been tested in major crop species yet. In this study, we constructed three vectors: pBeYDV-Cas9-KO, pRGEB32-GhU6.7, and pRGEB32-35S with the *GhCLA1* gene as the target, and analyzed their editing (knock out) efficiency in the cotton genome. We found that the pBeYDV-Cas9 vector mainly produced larger DNA fragment deletions, which is more likely to cause frameshift mutation for the target gene. The editing window of pBeYDV-Cas9-KO is wider when compared to pRGEB32-GhU6.7 and pRGEB32-35S vectors, ranging from 11th to 17th base at the 5’ end of the target site, with the highest editing frequency at both At and Dt subgenomes. Taken together, pBeYDV-Cas9-KO gene editing system presented stronger editing efficiency.

## 2. Materials and Methods

### 2.1. Construction of the Vectors

The pBeYDV-Cas9-KO vector was constructed from the pRGEB32-GhU6.7 vector as the template. The sgRNA sequences was constructed in the replicon of BeYDV geminivirus. First, two fragments of ULIR+Sites and BeSRL were obtained by gene synthesis. BeSRL is a sequence that continuously synthesizes the fragments of SIR, Rep, and DLIR. Then, the ULIR+Sites fragment inserted into the target vector pRGEB32-GhU6.7 was digested by Hind III restriction enzyme through the In-Fusion method (Vazyme, Nanjing, China). After sequencing correctly, it was named BeYDV-Cas9-ULIR vector. By using the same method, the BeSRL fragment was cloned into Sbf I-linearized BeYDV-Cas9-ULIR plasmid, it was named pBeYDV-Cas9-KO for cotton genome editing.

For the construction of pBeYDV-GFP/RFP expression vector, by using the pBeYDV-Cas9-KO vector as the template, 35S-GFP (CaMV 35S promoter) and 35S-RFP sequences were constructed in the replicon BeYDV geminivirus. pBeYDV-Cas9-KO was purified by MauB I and Sal I restriction endonuclease after digestion. The cauliflower mosaic virus (CaMV) 35S promoter has very high activity in a wide range of heterologous systems. The 35S promoter and *GFP/RFP* were linked to the vector by PCR and In-Fusion method. The 35S-GFP and 35S-RFP sequences were cloned into *MauB* I and *Sal* I linearized pBeYDV-Cas9-KO plasmids by In-Fusion method. After correct sequencing, they were named pBeYDV-GFP and pBeYDV-RFP, and then transformed into Agrobacterium for cotton transformation. 

For the construction of pBeYDV-GFP/RFP expression vector, by using the pRGEB32-GhU6.7 vector as the template [2], the Ubi promoter of *Cas9* in the vector was replaced by the 35S promoter and the 35S promoter was connected to pRGEB32-GhU6.7 vector by In-Fusion method. After correct sequencing, they were named pRGEB32-35S-KO for cotton genome editing.

In order to verify the editing efficiency of pBeYDV-Cas9-GhCLA1, pRGEB32-GhU6.7-GhCLA1, and pRGEB32-35S-GhCLA1 vectors in cotton, cotton chlorophyll synthesis gene *GhCLA1* was selected as the target gene because *GhCLA1* is responsible for chloroplast development of plant, and its multination will result in an albino phenotype in cotton leaves [2]. According to the CDS sequence of *GhCLA1* gene, six sgRNA sequences were designed and generated tRNA-gRNA tandem sequence (polycistronic tRNA-gRNA) as the target sequence, which were named as PTG1 (tRNA-sgRNA1-tRNA-sgRNA2), PTG2 (tRNA-sgRNA3-tRNA-sgRNA4), PTG3 (tRNA-sgRNA5-tRNA-sgRNA6). These three fragments were cloned into *Bsa* I-linearized pBeYDV-Cas9-KO, pRGEB32-GhU6.7 and pRGEB32-35S plasmids by In-Fusion assembly. Finally, we obtained pBeYDV-Cas9-GhCLA1, pRGEB32-GhU6.7-GhCLA1, and pRGEB32-35S-GhCLA1 genome editing vectors. These vectors were separately transformed into Agrobacterium tumefaciens strain GV3101 for cotton transformation. The primers used in vector construction are listed in Table S1.

### 2.2. Agrobacterium-Mediated Transformation of Cotton

Cotton cultivar *Gossypium hirsutum cv*. Jin668 [47] was used as the transformation receptor for *GhCLA1* gene editing using the three genome editing vectors in this study. Seeds of the receptor plants were sterilized and cultured in a modified solid MS medium [48] without light for 6 days at 30 °C. Hypocotyls were cut into 5–10 mm segments and used as explants for Agrobacterium-mediated transformation following our previous methods [7,48,49]. All the regenerated plants were grown in a tissue culture room with a 14 h light/10 h dark condition at 25 °C [2].

### 2.3. Fluorescence Microscopy Imaging

Cotton somatic embryos containing *GFP* and *RFP* genes were observed with Nikon stereomicroscope SMZ25 (Nikon, Tokyo, Japan). The green fluorescence was observed at an emission wavelength of 488 nm with an excitation wavelength of 490 nm to 520 nm. The red fluorescence was observed at an emission wavelength of 575 nm with an excitation wavelength of 530 nm to 550 nm. 

### 2.4. qRT-PCR

To determine the expression levels of *GFP* and *RFP* genes in the somatic embryos, qRT-PCR analyses were performed. The primers used in our study are listed in Supplemental Table S1. The relative expression level of *GFP* and *RFP* was estimated using the comparative threshold cycle (Ct) technique (2−ΔΔCt method) [50]. First-strand cDNA was generated from 3 mg of total RNA using SuperScript III reverse tran-scriptase (Invitrogen, Waltham, MA, USA). qRT-PCR was performed in 15 mL reactions using the ABI 7500 Real-Time PCR System (Thermo Electron Corporation, Waltham, MA, USA), with at least three technical replicates and three independent biological replicates. For each independent biological replicate, we took material samples from at least five samples of each vector. GhUB7 was used as an internal control. 

### 2.5. PCR Analysis and Sanger Sequencing 

Genomic DNA of the cotton plants were extracted with Plant Genome Extraction Kit (TIANGEN, Beijing, China). For transgenic positivity check, specific primers of *Cas9* sequence were used in PCR analysis. Partial *GhCLA1* sequences that covered transgenic sgRNA1-sgRNA2 sites, sgRNA3-sgRNA4 sites, sgRNA5-sgRNA6 sites, and their predicted off-target sites, respectively, were used for mutation genotyping in independent lines. The obtained PCR products were ligated in pGEMT-Easy vector for TA cloning with T4 DNA ligase (Promega, Madison, WI, USA). After reaction, the ligated products were transformed into E. coli strain Top10 and positive clones were applied for DNA Sanger sequencing.

### 2.6. On-Target and Off-Target Mutation Analysis by Hi-Tom High-throughput Sequencing and Sanger Sequencing

For transgenic plants, according to the number of positive plants to be detected, Hi-Tom (High-throughput Tracking of Mutations) primers were designed to amplify the target sequence for high-throughput sequencing [51]. First, primers were designed at 150 bp at both ends of the sgRNA site, the target sequence was amplified by two rounds of PCR, and the editing of the target sequence was analyzed by deep sequencing, a pair of different barcodes on the front and back primers correspond to different samples. We can find the corresponding samples through the labels and analyze the editing of their sequences. The primers used in our study are listed in Supplemental Table S1. Independent samples were amplified by PCR using corresponding barcode primers, and the resulting PCR products were equally mixed and purified (Magen, Guangzhou, China). The DNA fragments were sequenced by Illumina and analyzed via the Hi-Tom website (http://www.hi-tom.net/hi-tom/ (accessed on 20 May 2022)).

Based on the off-target prediction software [52], five predicted off-target sites with the highest scores corresponding to sgRNA1–sgRNA5 were found from the cotton genome. Predicting off-target *E. coli* strain Top10 and positive clones were applied for DNA Sanger sequencing.

## 3. Results

### 3.1. The BeYDV Replicon showed High Activity in Cotton Cells Visualized by GFP and RFP Reporter Genes

**Figure 1:**
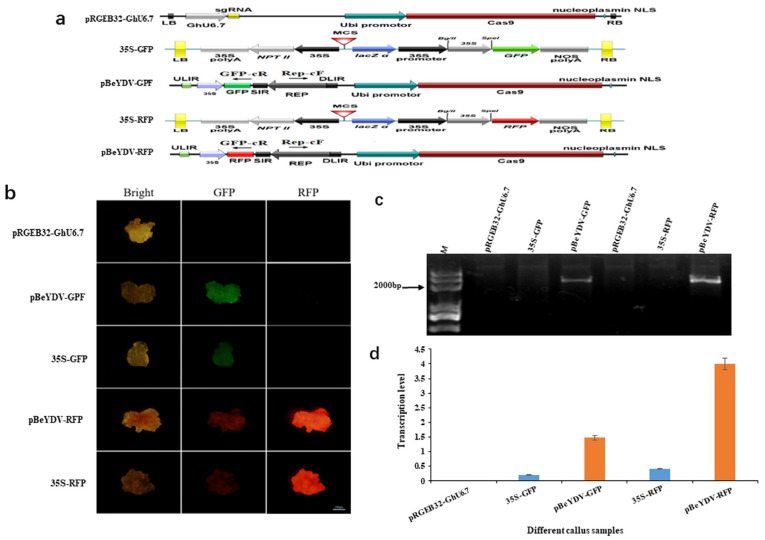
The analysis of *GFP/RFP* activity within the BeYDV. (**a**) The schematic diagram of pBeYDV-GFP/RFP and 35S-GFP/RFP vectors in cotton. RB and LB are Agrobacterium right and left borders. ULIR, DULIR: large intergenic region; SIR: small intergenic region; Rep: replication protein. (**b**) *GFP* and *RFP* expression in cotton callus with different vectors. The cotton callus targeting to *GFP* and *RFP* fluorescence. The fluorescence of callus transformed with pBeYDV-GFP and pBeYDV-RFP vectors was significantly stronger than 35S-GFP and 35S-RFP vectors. pRGEB32-GhU6.7: The control. Bar = 0.5 cm. (**c**) Detection of cyclized structure of BeYDV in cotton callus. The DNA samples transformed with pBeYDV-GFP and pBeYDV-RFP vectors can amplify a band with the size of 2.1 kb using specific primers (Rep-cF and GFP/RFP-cR). (**d**) The relative expression of *GFP* and *RFP* in pRGEB32-GhU6.7, 35S-GFP, 35S-RFP, pBeYDV-GFP, and pBeYDV-RFP cotton callus.

In order to verify whether replicon of BeYDV can improve transgene expression in cotton genome, four vectors (35S-GFP, 35S-RFP, pBeYDV-GFP, and pBeYDV-RFP) were designed and transformed into cotton genome through Agrobacterium mediated genetic transformation. The *GFP* and *RFP* genes were inserted into the replicon of BeYDV geminivirus and named as pBeYDV-GFP and pBeYDV-RFP. The pRGEB32-GhU6.7 vector was used as control (empty) vector to transfer into cotton at the same time (Figure 1a). To check whether BeYDV vector can be intergraded into cotton genome, we designed two pairs of primers that can specifically amplify the fragment in the cyclized fragment to detect whether BeYDV is cyclized in cotton genome. Upstream and downstream primers were designed from the BeSRL and *GFP/RFP* regions, the length of the amplified fragment was 2.1 kb. The result shows that a 2.1 kb band can be amplified from the transgenic calli containing pBeYDV-GFP and pBeYDV-RFP vectors, and no target band was detected in the samples transformed with pRGEB32-GhU6.7, 35S-GFP, and 35S-RFP vectors (Figure 1c). These results suggested that pBeYDV-GFP and pBeYDV-RFP vectors could form cycle structure for replication in cotton genome.

In order to verify the transcription level of *GFP* and *RFP* in pBeYDV replicon, RNA was isolated from transgenic cotton calli containing pRGEB32-GhU6.7, 35S-GFP, 35S-RFP, pBeYDV-GFP, and pBeYDV-RFP and qRT-PCR data revealed that the transcription of *GFP* and *RFP* in the pBeYDV-GFP and pBeYDV-RFP calli was 7.7 times and 9.7 times higher than in the 35S-GFP and 35-RFP calli, respectively (Figure 1d). In addition, when compared the fluorescence in the transgenic calli with pBeYDV-GFP and pBeYDV-RFP vectors, they were obviously stronger than the calli with 35S-GFP, 35S-RFP vectors (Figure 1b). These data suggest that the replicon of BeYDV can dramatically improve the transcription level of target genes in cotton genome (Figure 1d), which provides solid basis for the further utilization of BeYDV vector for genome editing in cotton.

### 3.2. Design of Genome Editing Vector Based on the Replicon of BeYDV and Comparison of Editing Efficiency with Other Vectors

**Figure 2:**
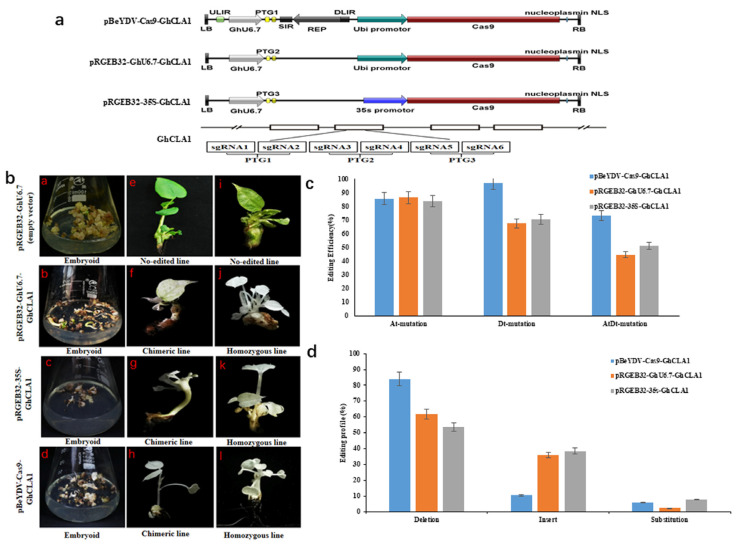
Comparison of editing efficiency of different genome editing vectors in cotton. (**a**) The schematic diagram of pBeYDV-Cas9-GhCLA1, pRGEB32-GhU6.7-GhCLA1, and pRGEB32-35S-GhCLA1 vectors. GhCLA1: Target gene. PTG: Polycistronic tRNA-gRNA, PTG1: tRNA-sgRNA1-tRNA-sgRNA2, PTG2: tRNA-sgRNA3-tRNA-sgRNA4, PTG3: tRNA-sgRNA5-tRNA-sgRNA6. (**b**) Phenotype of the T0 cotton plants with the target mutations in GhCLA1 gene. a, b, c, d: Callus materials of different vectors; e, i: The leaves of T0 plants (pGREB32-GhU6.7) exhibited green phenotype; the leaves of T0 plants (pBeYDV-Cas9-GhCLA1, pRGEB32-GhU6.7-GhCLA1, and pRGEB32-35S-GhCLA1) exhibited chimeric phenotype (**f**, **g**, **h**) and albino phenotype (**i**, **k**, **l**) Bar = 1 cm. (**c**) AtDt Mutation frequencies of three vectors with GhCLA1 sgRNAs in cotton T0 lines. The efficiency of simultaneously editing At and Dt subgenomes is higher by pBeYDV-Cas9-GhCLA1 vector. Mutation frequencies in stable transgenic T0 lines. Each line was detected by Hi-Tom sequencing of PCR amplicons. (**d**) Comparison target mutation profiles of T0 plants edited by three systems. The editing types of target genes of transgenic plants with different vectors include fragment deletion, base insertion and base replacement. The editing type of the transgenic plants of pBeYDV-Cas9-GhCLA1 vector is mainly fragment deletion, accounting for 84.0%. The fragment deletion accounted for 61.8% and 53.8% in the transgenic plants of pRGEB32-GhU6.7-GhCLA1 and pRGEB32-35S-GhCLA1 vectors, respectively. Mutation types in stable transgenic T0 lines. Each line was detected by Hi-Tom sequencing of PCR amplicons.

As shown previously, the replicon of BeYDV can drive high lever expression of foreign genes in cotton cells. Therefore, we introduced the replicon of BeYDV into pRGEB32-GhU6.7 and generated a novel genome editing vector pBeYDV-Cas9-KO. Meanwhile, the cotton Ubi promotor in the pRGEB32-GhU6.7 vector was replaced with CaMV 35S promoter to obtain the pRGEB32-35S vector. In order to verify the editing efficiency of these vectors in cotton genome, *GhCLA1* gene was selected as the target gene. Three vectors used to genetic transformation were constructed and named as pBeYDV-Cas9-GhCLA1, pRGEB32-GhU6.7-GhCLA1, pRGEB32-35S-GhCLA1 (Figure 2a) and transformed them into cotton cells by *Agrobacterium*-mediated genetic transformation. 

Compared with the transgenic plant with pRGEB32-GhU6.7 empty vector (without sgRNA), the T0 plants with other three vectors exhibited chimeric or complete albino phenotype (Figure 2b). For the pRGEB32-GhU6.7-GhCLA1 vectors, 32 T0 plants were regenerated including 11 chimeric plants and 21 complete albino plants, and we obtained 42 T0 plants with pRGEB32-35S-GhCLA1 vectors, including 13 chimeric plants and 29 complete albino plants. Meanwhile, we obtained 51 positive plants, including 5 chimeric plants and 46 complete albino plants with pBeYDV-Cas9-GhCLA1 vector suggesting that this system exhibited highest (up to 90%) editing efficiency compared with pRGEB32-GhU6.7-GhCLA1 and pRGEB32-35S-GhCLA1 vectors. In order to accurately evaluate the editing efficiency of these three vectors, Hi-Tom high-throughput sequencing was used to detect the target editing efficiency. The results showed that the efficiency of simultaneous editing of *GhCLA1* gene in At and Dt subgenomes with pBeYDV-Cas9-GhCLA1 vector was up to 73.3%, while the efficiency of vector pRGEB32-GhU6.7-GhCLA1 and pRGEB32-35S-GhCLA1 was 44.6% and 51.2%, respectively (Table S2). The editing efficiency in of *GhCLA1* gene in At or Dt subgenome with pBeYDV-Cas9-GhCLA1 vector was 85.7% and 97.2%, respectively. The editing efficiency of pRGEB32-GhU6.7-GhCLA1 and pRGEB32-35S-GhCLA1 vectors was 86.5%, 67.7%, 84%, and 70.5%, respectively (Supplemental Table S2) (Figure 2c). These data showed that pBeYDV-Cas9 system exhibited higher editing efficiency in cotton genome, especially can create homozygous mutations with the alleles in At and Dt subgenomes edited simultaneously. 

### 3.3. Comparison of Editing Profile of Different Genome Editing Vectors in Cotton

**Figure 3:**
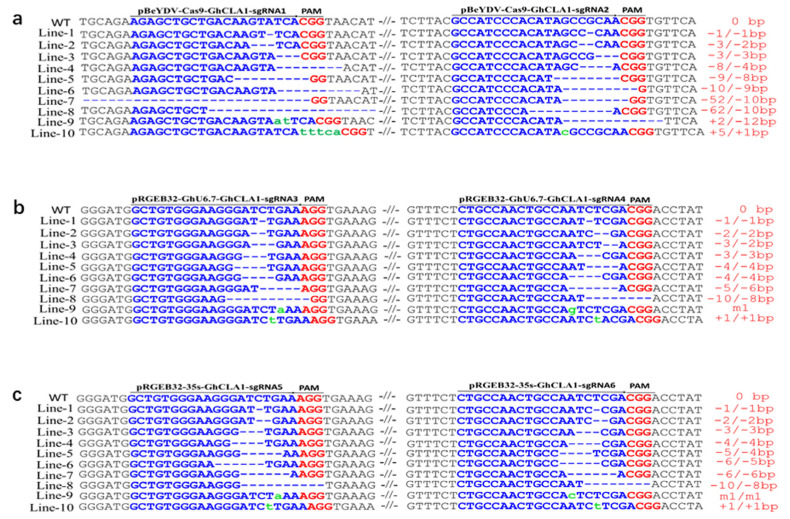
Comparison target mutation sequences of T0 plants edited by three vectors. (**a**): The target mutation profiles of T0 plants edited by pBeYDV-Cas9-GhCLA1. (**b**): The target mutation profiles of T0 plants edited by pRGEB32-GhU6.7-GhCLA1. (**c**): The target mutation profiles of T0 plants edited by pRGEB32-35S-GhCLA1.

DSBs generated by CRISPR/Cas endonuclease cleavage are mainly repaired by NHEJ (non-homologous end joining) pathway in plant cells. In the process of NHEJ, due to different target specificity and Cas endonuclease, they usually show differences in editing profiles. The Hi-Tom sequencing data revealed that the editing profile of target genes included DNA fragment deletion, insertion, and single base transversion and transition. The editing profile of pBeYDV-Cas9-GhCLA1 is mainly composed of large DNA fragment deletion, accounting for 84.0% with the deletion, and insertion, base substitution accounted for 10.4% and 5.7%, respectively. The deletion accounted for 61.8% and 53.8%, insertion accounted for 36.0% and 38.5%, and the base substitution accounted for 2.2% and 7.7% in the plants harboring pRGEB32-GhU6.7-GhCLA1 and pRGEB32-35S-GhCLA1 vectors, respectively (Figure 2d and Figure 3, Table S3). These data show that the editing profile of pBeYDV-Cas9-GhCLA1 in cotton is mainly composed of large fragment deletion (Figure 2d and Figure 3), which can more completely mutate the target genes of cotton. The reason for large DNA fragment deletion of pBeYDV-Cas9-GhCLA1 may be that there are abundant sgRNAs at the target sites, which could create more DNA cutting and then boost the repair of NHEJ in plant cells.

**Figure 4:**
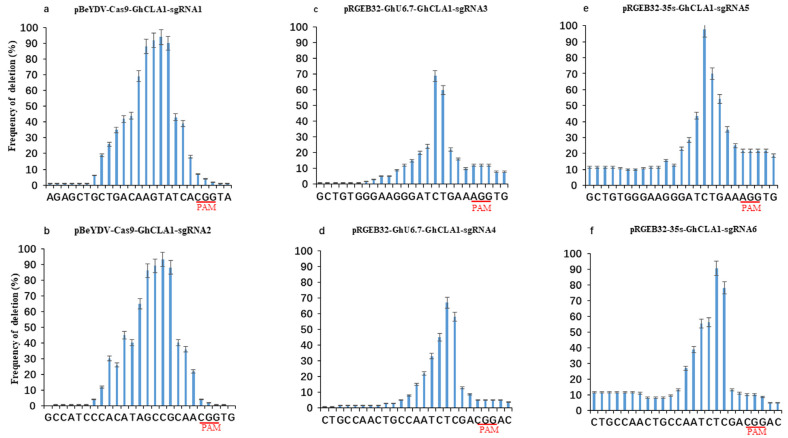
Comparison of positional deletion frequencies by three systems. (**a**): pBeYDV-Cas9-GhCLA1-sgRNA1. (**b**): pBeYDV-Cas9-GhCLA1-sgRNA2. (**c**): pRGEB32-GhU6.7-GhCLA1-sgRNA3. (**d**): pRGEB32-GhU6.7-GhCLA1-sgRNA4. (**e**): pRGEB32-35S-GhCLA1-sgRNA5. (**f**): pRGEB32-35S-GhCLA1-sgRNA6. PAM is underline. The experiments were carried out in cotton T0 plants and frequencies were measured by Hi-Tom sequencing.

**Figure 5:**
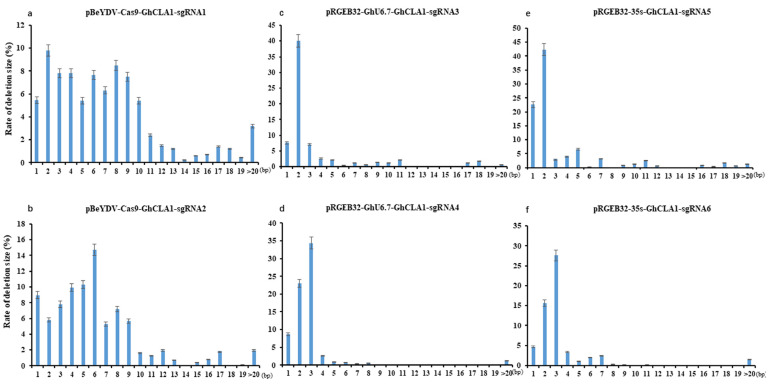
Frequencies of deletions of different sizes at different target sites by three different systems. (**a**): pBeYDV-Cas9-GhCLA1-sgRNA1. (**b**): pBeYDV-Cas9-GhCLA1-sgRNA2. (**c**): pRGEB32-GhU6.7-GhCLA1-sgRNA3. (**d**): pRGEB32-GhU6.7-GhCLA1-sgRNA4. (**e**): pRGEB32-35S-GhCLA1-sgRNA5. (**f**): pRGEB32-35S-GhCLA1-sgRNA6. PAM is underline. The experiments were carried out in cotton T0 plants and frequencies were measured by Hi-Tom sequencing.

In order to more precisely analyze the editing window at the target sites, we counted the editing location of each base through high-throughput sequencing. The results show that the mutation at 4–6 bp upstream of PAM has the highest frequency (Figure 4). However, the editing sites of pBeYDV-Cas9-GhCLA1 vector are mainly located at positions 4–10 upstream of PAM site (Figure 4a,b). Whereas the editing sites of pRGEB32-GhU6.7-GhCLA1 vector are mainly located at positions 4–6 upstream of PAM site (Figure 4c,d), the editing sites of pRGEB32-35S-GhCLA1 vector are mainly located at positions 3–6 upstream of PAM site (Figure 4e,f). In addition, we analyzed the length of the deletion fragment in the mutant sequence of the target gene *GhCLA1* of the three vectors. In the pBeYDV-Cas9-GhCLA1 plants, the deletion size is larger compared to the other two vectors, the deletions size ranging from 8 to 62 bp accounted for 33.93% of all deletion types (Figure 5a,b), and the deletion size mainly ranges from 1 bp to 3 bp in the pRGEB32-GhU6.7-GhCLA1 plants and pRGEB32-35S-GhCLA1 vectors, which accounted for 83.76% and 76.65% of all deletion types and the large size deletions (8 bp) accounted for 8.03% and 8.01% of all deletion types, respectively (Figure 5c,d,e,f). Therefore, pBeYDV-Cas9-GhCLA1 genome editing system has a wider editing window at target site editing in cotton genome with a larger size deletion.

### 3.4. The off-Target Effect Analysis of Different Genome Editing Vectors in Cotton

**Figure 6:**
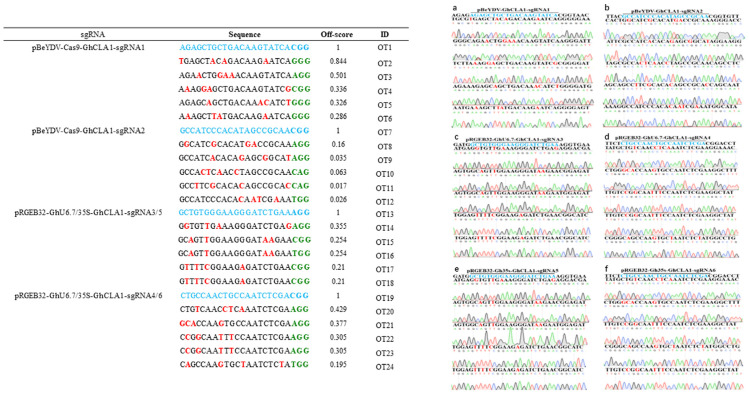
The information of predicted off-target sites and analysis of potential off-target effect in T0 plants using three systems. Off-target effects were detected at predicted potential off-target sites in independent T0 plants by Sanger sequencing. Five predicted potential off-target sites were selected for each target. Mismatching bases are shown in red; the PAM motifs are shown in green. Off-target effects were detected at five predicted potential off-target sites in six independent T0 plants by Sanger sequencing. pBeYDV-Cas9-GhCLA1 (**a**,**b**), pRGEB32-GhU6.7-GhCLA1 (**c**,**d**), pRGEB32-35S-GhCLA1 (**e**,**f**).

CRISPR/Cas9 genome editing system can produce efficient and targeted mutations in cotton; however, there are many highly homologous alleles in this tetraploid genome. These homology sequences will also have the probability of being edited and this kind of unwanted editing is called off-target editing [15,33,47]. Based on the off-target prediction software [52], five predicted potential off-target sites with the highest scores corresponding to sgRNA1–sgRNA5 were identified in the cotton genome (Figure 6). Most of these predicted off-target sites are located in the non-coding region of the genome. Therefore, we selected the conventional PAM type of 5’-NGG-3’ for further analysis. The Figure 6 presents predicted off-target sites of different sgRNA, the off-target effect scores, the starting sites in the genome, and the off-target sequences located in the CDS region or noncoding region. Sanger sequencing method was used to detect the predicted off-target sites and compared them with the corresponding reference sequences. The results showed that there were no mutations at these predicted off-target sites corresponding to sgRNA target sites of three genome editing vectors (Figure 6). 

## 4. Discussions

CRISPR/Cas genome editing technology has become the routine means for plant functional genomics and molecular breeding research. At present, CRISPR/Cas9 genome editing technology has been used to edit target genes by insertion, deletion, base substitution through NHEJ [20]. It has been well applied in tetraploid upland cotton [2] as well. However, the editing efficiency in cotton is still low because of the gene redundancy and the complex genome. Geminivirus-derived vectors can produce enough donor templates by rolling-cycle replication after infecting plant cells, and they can replicate and express rapidly in organisms without random insertion into plant genomes, and will not cause toxic effects on plant organisms [53]. It can increase the expression level of donor templates by rolling-circle replication combining the large intergenic region (LIR), the short intergenic region (SIR), and the trans-acting replication initiation protein (Rep). Based on these, some studies have shown that the method of combining geminivirus vector with CRISPR-Cas9 technology can increase genome editing efficiency in plants [41,53,54]. Vu shows the highly efficient HDR in tomato using CRISPR/Cpf1 system combined with geminiviral replicon [4]. The use of geminivirus system can expand the application range of plant genome editing system. However, this system has not been tested in the major crop species, therefore, we developed the first update CRISPR/Cas9 system-pBeYDV-Cas9-KO based on the geminivirus replicon in cotton.

Geminivirus infects a wide variety of plants, including monocotyledons and dicotyledons. Because movement and coat proteins are essential for viral transmission, they were replaced with other safe DNA fragments. The modified virus is not capable of transmission and infection due to the lack of essential viral proteins. No biosafety concern with the use virus-enhancing transcription/expression sequencing was presented in the previous reports, and in case of field release recombination any concern may have been prevented by comparing the inserted virus sequences with sequences of the virus naturally infecting cotton in the region by in silico analysis. Different geminivirus hosts are different, and only by selecting suitable geminivirus vectors they can be active in hosts and perform further gene editing in plants. Wang used wheat dwarf virus (WDV) in rice [54], but it does not work with cotton (Date did not show). The cabbage leaf curl virus (CaLCUV) has been used in arabidopsis and the bean yellow dwarf virus (BeYDV) has been used in tobacco and tomato [41,55]. In order to verify the efficiency of geminivirus-mediated gene editing vector in cotton, first, the expression activities of BeYDV geminivirus replicon were tested in cotton cells, which proved that BeYDV geminivirus vector can be expressed in cotton genome and can improve the quantity of donor template (sgRNAs). Compared to the two genome editing vectors of pRGEB32-GhU6.7 and pRGEB32-35S, the editing profile of sgRNA using pBeYDV-Cas9-KO vector showed some differences although the editing site is mainly located 3 bp upstream of PAM site. The DNA deletion size is larger and homozygous editing efficiency is much higher, which may be explanted that in the process of gene editing, explained by the fact that the replicon of BeYDV geminivirus increase the transcription of sgRNAs, which could guide more Cas9 to locate the target sites. Under the endonuclease action of Cas9 protein with sufficient amount, the frequency of cutting target sites is greatly increased, therefore the editing efficiency is improved, then the proportion as well as size of DNA deletion at the target site is greatly improved. The deletion length of large fragments can reach 62 bp in this report due to the high amount of sgRNAs transcripted from the replicon of BeYDV geminivirus, which exhibits great potential to be used for the large DNA fragments deletion in plant cell. The large DNA fragments deletion can cause partial deletion of chromosomes or delete the whole chromosome, which may change spatial structure of chromatin and create excellent varieties [56].

Overall, the editing efficiency of pBeYDV-Cas9-KO is higher than previous genome editing system in cotton which can expand the development of cotton genome editing system. At the same time, in order to optimize the gene editing efficiency mediated by BeYDV geminivirus, the replicon of BeYDV geminivirus could be combined with CRISPR-Cpf1 system to expand the scope of genome editing by designing gRNAs against various targets with different PAM sites. CRISPR/Cas9-BeYDV replicon genome editing system is expected to provide a more powerful tool for cotton functional genomics research and molecular breeding.

## Data Availability

The templated vectors and destination vector will be available at Addgene.

## Supplementary Materials

The following are available online at www.mdpi.com/article/10.3390/cells11182902/s1, Additional Supporting Materials may be found online in the supporting information tab for this article. Table S1: Primers used for vector construction and transgenic plant detection. Table S2: Mutation Frequencies of three vectors with *GhCLA1* sgRNAs in cotton T0 lines. Table S3: Statistics of mutation profiles using three systems in T0 generation plants.
